# Safe Sedation and Analgesia Management With Remifentanil in Pediatric ICU Patients: A Report of Two Cases

**DOI:** 10.7759/cureus.79435

**Published:** 2025-02-22

**Authors:** Mana Tamaki, Takahiro Hirai, Tasuku Fujii, Shogo Suzuki, Takahiro Tamura

**Affiliations:** 1 Department of Anesthesiology, Yokkaichi Municipal Hospital, Yokkaichi, JPN; 2 Department of Anesthesiology, Nagoya University Hospital, Nagoya, JPN; 3 Department of Anesthesiology, Nagoya University Graduate School of Medicine, Nagoya, JPN

**Keywords:** analgesia, intensive care unit, pediatric, remifentanil, sedation

## Abstract

Although remifentanil (RF) is not approved for use in the ICU for children, its short half-life and titratability may provide advantages in children requiring high doses of sedatives and analgesics during prolonged mechanical ventilation. This report presents two pediatric cases in which RF was administered for sedation and analgesia, enabling successful extubation without adverse effects.

In Case 1, a four-year-old girl with multiple atypical teratoid rhabdoid tumors required mechanical ventilation due to impaired consciousness. RF was initiated at 0.22 μg/kg/min and titrated up to 0.48 μg/kg/min on the day before extubation, allowing the reduction of other sedatives. She received RF for a total of 16 hours (5.66 mg) before it was discontinued, and extubation was successfully performed 70 minutes later.

In Case 2, a three-year-old girl with a severe neck abscess required prolonged intubation following surgical drainage. RF was started at 0.1 μg/kg/min and increased to 0.2 μg/kg/min on the day before extubation, facilitating the tapering of other sedatives. She received RF for a total of 23 hours (4.4 mg) before discontinuation, and extubation was successfully performed 25 minutes later.

Throughout RF administration, hemodynamic stability was maintained, with no occurrences of hypotension, bradycardia, desaturation, hepatic or renal dysfunction, muscle rigidity, or hyperalgesia. No additional catecholamines were required, and no signs of withdrawal symptoms were observed. These findings suggest that RF may be a safe and effective option for sedation and analgesia in pediatric patients undergoing prolonged mechanical ventilation.

## Introduction

Achieving optimal sedation and analgesia in pediatric patients on mechanical ventilators is challenging due to limited verbal communication and the need for deep sedation to avoid self-extubation and removal of catheters [1]. Propofol, a short-acting anesthetic/sedative commonly used in adults, is not approved in Japan for use in children in the intensive care unit (ICU) due to side effects such as rhabdomyolysis and high mortality associated with long-term sedation [2]. In Japan, midazolam (MDZ) and fentanyl (FNT) are typically used for pediatric patients. However, the clearance of these drugs varies depending on the patient's age and liver function, potentially leading to prolonged sedation. Furthermore, these drugs exhibit extended context-sensitive half-times, and prolonged continuous administration can significantly increase their half-life, potentially complicating weaning from mechanical ventilation during long-term sedation in the ICU [3]. Discontinuation of these medications may lead to withdrawal symptoms, including agitation, anxiety, muscle tension, diarrhea, fever, sweating, and tachypnea in pediatric patients [4]. The occurrence of withdrawal syndrome can complicate the prediction of the optimal timing for weaning from mechanical ventilation.

Remifentanil (RF) is rapidly hydrolyzed by nonspecific esterases in blood and tissues [5], unaffected by liver or kidney function, allowing for rapid titration of both analgesic and anesthetic depth with minimal fluctuations in hemodynamics [6]. With an ultra-short half-life of about three to four minutes, RF offers rapid onset and offset, making it highly adjustable, even for long-term use or high doses. Although widely used in ICUs worldwide, as of November 2024, RF remains unapproved for analgesia in pediatric ICUs [7-10].

In this report, we describe two pediatric cases where off-label use of RF provided safe and effective sedation and analgesia in the ICU. These cases highlight the potential role of RF in pediatric critical care and underscore its advantages in overcoming some of the limitations of conventional sedatives. Considering Japan's rigorous emphasis on drug safety, these findings may serve as important evidence supporting the future approval of RF for use in pediatric ICUs.

This article was previously presented as a meeting abstract at the 51st Annual Meeting of the Japanese Society of Intensive Care Medicine on March 16, 2024.

## Case presentation

The off-label use of RF and the presentation of this study were submitted to and approved by the unapproved new drug evaluation committee and the bioethics review committee of our institution. We obtained written consent from the family for the off-label use of RF.

Case 1

**Figure 1:**
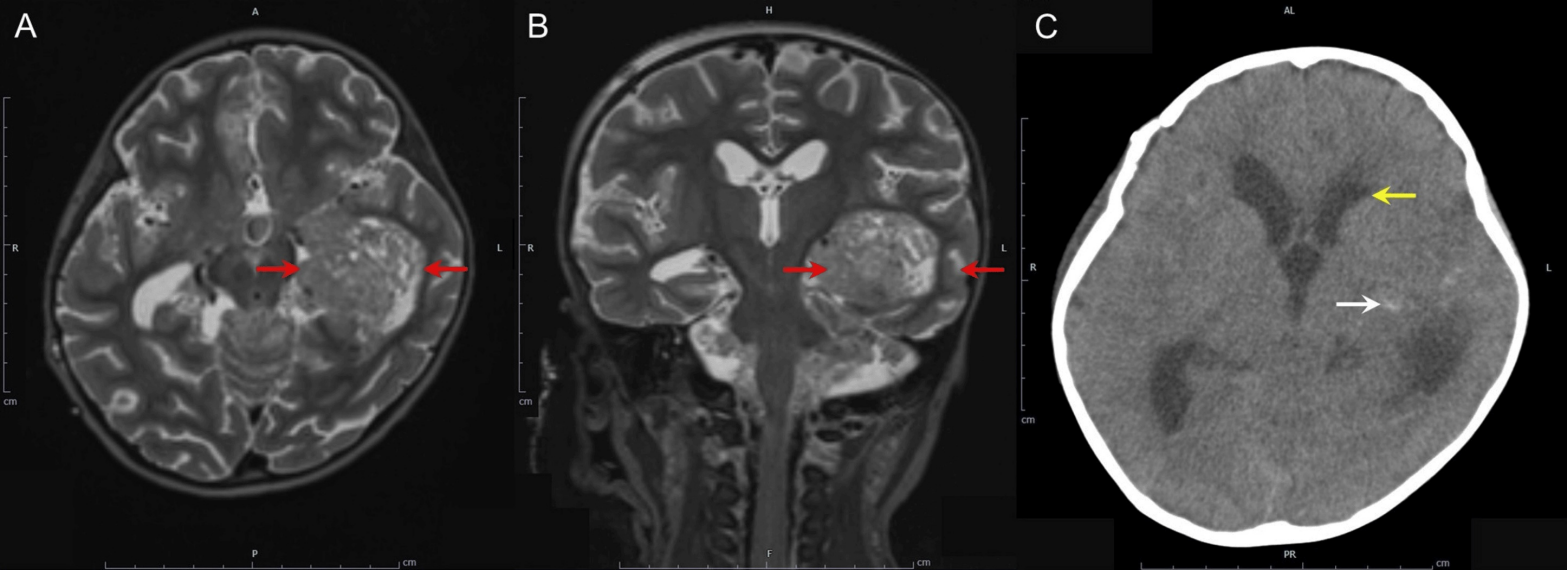
MR FLAIR and CT images of the brain in Case 1. Axial (A) and coronal (B) MR FLAIR images showing a mass lesion with mild heterogeneous hyperintensity in the left temporal lobe and lateral ventricle (red arrow); (C) CT image showing narrowing of the lateral ventricles (yellow arrow) and intratumoral hemorrhage (white arrow). MR: magnetic resonance; CT: computed tomography; FLAIR: fluid-attenuated inversion recovery

A four-year-old girl, height 96 cm, weight 15 kg, undergoing radiochemotherapy for multiple atypical teratoid rhabdoid tumors in the left temporal lobe and lateral ventricle (Figures 1A-1B), was admitted to the ICU due to vomiting and generalized convulsions. At admission, the convulsions had ceased, but she had a Glasgow Coma Scale score of 6 (E4V1M1) and impaired consciousness. Respiratory and circulatory stability was maintained with 5 L/min oxygen via a mask. A head CT showed narrowing of the lateral ventricles and intratumoral bleeding (Figure 1C). The medical team, including a brain surgeon and anesthesiologist, decided to intubate her until her condition improved. MDZ, dexmedetomidine (DEX), and FNT were administered with a goal of Richmond Agitation-Sedation Scale (RASS) score of -3, with doses increased if she moved. Levetiracetam 150 mg and lacosamide 45 mg were administered, and she had no convulsions after admission. A head MRI on day 4 showed no new bleeding and improved cerebral edema, leading to the decision to extubate on day 5. The cuff leak test was positive, and furosemide 5 mg was administered every six hours to promote diuresis, and dexamethasone 6.6 mg was administered six times every six hours to prevent laryngeal edema.

**Figure 2:**
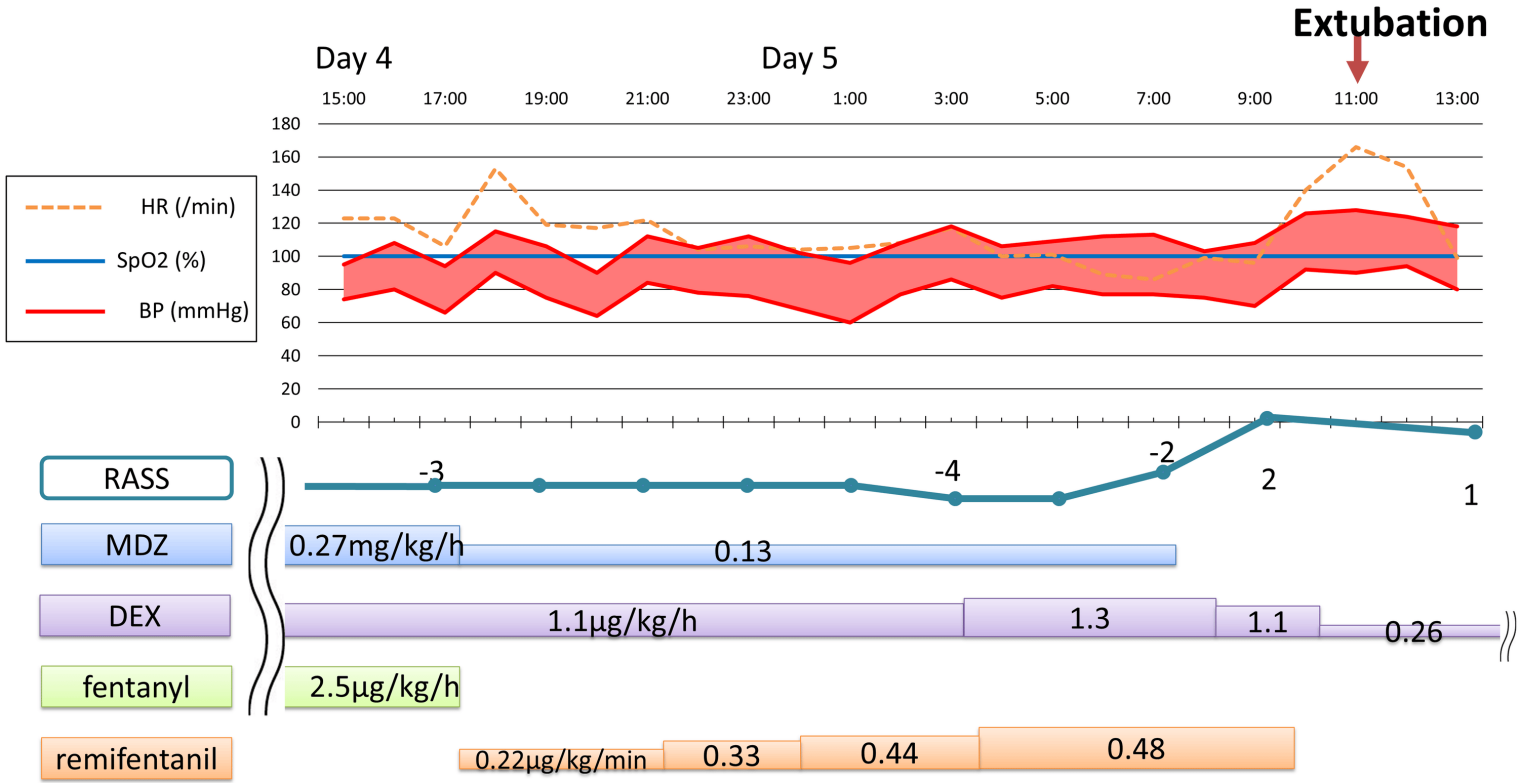
Clinical course of Case 1. RASS: Richmond Agitation-Sedation Scale; MDZ: midazolam; DEX: dexmedetomidine

Off-label use of RF for analgesia and sedation was initiated on day 4 at a dose of 0.22 μg/kg/min, based on her weight, while other sedative medications were gradually tapered or discontinued to prevent withdrawal symptoms (Figure 2). We increased the RF dose to 0.48 μg/kg/min whenever she exhibited body movements, while maintaining a RASS of -3. After RF was discontinued on day 5, the patient quickly woke up, and a successful spontaneous breathing trial (SBT) was completed. The medical team extubated the patient approximately 70 minutes after discontinuing RF. RF was used for 16 hours and the total dose was 5.66 mg. The patient progressed well and was discharged from the ICU on day 9.

Case 2

**Figure 3:**
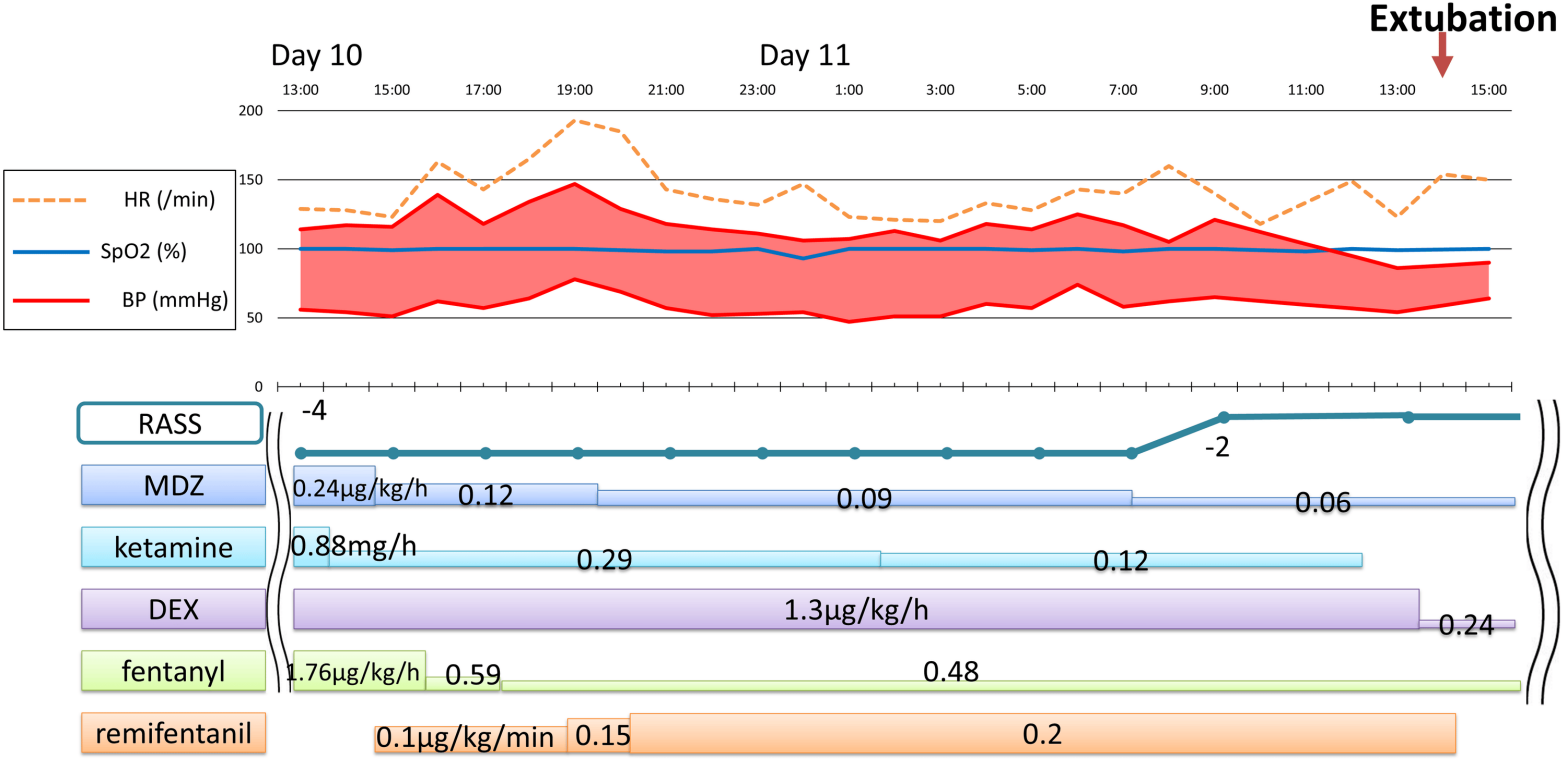
Clinical course of Case 2. RASS: Richmond Agitation-Sedation Scale; MDZ: midazolam; DEX: dexmedetomidine

One week after contracting impetigo, a three-year-old girl, height 97 cm, weight 17 kg, developed worsening swelling under her jaw despite antibiotics treatment. She was transferred to our hospital for drainage. After neck abscess drainage in the operating room, significant swelling led to ICU admission with continued intubation. MDZ, ketamine, DEX, and FNT were administered to achieve a RASS score of -4, with doses increased or additional doses given if the patient moved. At admission, ampicillin sodium and sulbactam sodium 1.125 g were administered every six hours, but on the third day, methicillin-resistant *Staphylococcus aureus* was detected in the abscess culture, prompting a switch to vancomycin. As neck swelling improved, extubation was planned for the 11th day of admission. On day 10, RF was started at 0.1 μg/kg/min, and MDZ, ketamine, and FNT were gradually tapered (Figure 3). As RASS remained at -4, RF was increased to 0.2 μg/kg/min based on the patient's body movements, with additional sedatives as needed. On day 11, spontaneous breathing occurred five minutes before RF was discontinued, and SBT was successful. Extubation occurred 25 minutes after RF discontinuation, following increased body movements. RF was used for 23 hours with a total dose of 4.4 mg. To prevent withdrawal syndrome, MDZ and FNT were continued at low doses even after extubation. On day 12, their doses were halved to MDZ 0.03 mg/kg/h and FNT 0.24 μg/kg/h. The patient improved and was discharged from the ICU on day 13 while continuing these two drugs. Over the course of three to four days, the MDZ was reduced by 0.1 mg/day and the FNT dose by 1 μg/day before discontinuation.

The drug dosage and vital signs before and after RF administration are shown in Figures 2-3, and there was no change in hemodynamics after RF. Additionally, as shown in Table 1, no new catecholamines were required after RF administration, and no deterioration in oxygenation, liver or kidney dysfunction, muscle stiffness, hyperalgesia, or withdrawal symptoms were observed.

**Table 1:** Key parameters before and after remifentanil administration and after extubation. P/F: PaO2/FiO2 ratio; AST: aspartate aminotransferase; ALT: alanine transaminase; Cre: creatinine

Case 1	Vasopressor	P/F ratio (mmHg)	AST/ALT (U/L)	Urine volume (mL/kg/day)	Serum Cre (mg/dL)
Before remifentanil	None	529	24/39	108	0.25
After remifentanil	None	529	24/34	76	0.31
After extubation	None	558	25/31	36	0.23
Case 2					
Before remifentanil	None	277	20/11	78	0.33
After remifentanil	None	277	20/11	92	0.24
After extubation	None	None (SpO_2_≧97%)	28/13	53	0.32
Reference range	-	400-500	24-43/9-30	-	0.2-0.4

## Discussion

We report two cases in which RF was used from approximately 24 hours before planned extubation until extubation in children who had been mechanically ventilated for several days in the ICU. After the start of RF, other sedatives and analgesics were either tapered or discontinued while maintaining appropriate sedation, allowing for early extubation RF administration. Importantly, no significant side effects were observed during their ICU stay. These cases highlight that RF can prevent withdrawal symptoms caused by long-term administration of benzodiazepines and help predict extubation timing.

In Japan, reports on RF use as an analgesic in children and newborns outside of surgical procedures are sparse, as RF is not yet approved in pediatric ICUs. One report [11] involved a non-intubated one-year-old, where RF was administered based on pain levels alongside DEX and FNT while monitoring the partial pressure of expired CO_2_. During RF use, SpO_2_ was 100%, the partial pressure of expired CO_2_ was ≤45 mmHg, and no respiratory issues, laryngeal spasms, or glottic closure were observed. However, RF use is only guaranteed to be safe under mechanically ventilated general anesthesia with sufficient monitoring, a medical safety system, and continuous monitoring by an anesthesiologist, and strict caution is required when using RF in non-intubated settings.

A randomized controlled trial (RCT) [5] reported that extubation time was significantly shorter in the RF group than in the FNT group. RF is also used to predict extubation timing, allowing experienced staff to extubate during the day, which is particularly useful for patients with difficult airways.

RF side effects include hypotension, bradycardia, deterioration of oxygenation due to laryngospasm and glottic closure [12], liver dysfunction, oliguria [13], muscle rigidity, and opioid-induced hyperalgesia (OIH) [14]. However, none of these adverse events occurred in our cases (see Table 1). In Welzing et al.’s RCT [15], the Children's and Infants' Postoperative Pain Scale (CHIPPS) Scale [16] was used to assess pain and detect OIH in infants. While we did not quantitatively evaluate OIH, the absence of tachycardia or elevated blood pressure suggested no hyperalgesia or OIH. Although the mechanism of OIH caused by RF remains unclear, it is more likely in adults with high doses of about 0.4 μg/kg/h [17] or long-term administration [18], and ketamine [16] or long-acting analgesic might help prevent it [7]. In Case 1, a high dose of RF was administered, but we believe high doses should be avoided even in the ICU. RF was used for a short period, starting 24 hours before the planned extubation, to minimize the risk of OIH and resistance. Withdrawal symptoms, such as tachycardia, tachypnea, sweating, and agitation, are commonly observed during the tapering of MDZ and FNT in long-term intubated children prior to extubation. However, none of these symptoms occurred in the present cases.

Conventional sedation management without RF often results in increased patient movement during the tapering of MDZ and FNT, necessitating continuous nursing observation to prevent accidental extubation. By providing more stable analgesia, RF may help alleviate this burden on nursing staff.

The cost of sedative medications in the pediatric ICU is approximately $12 per day for FNT and about $28 per day for RF. Although RF is more expensive, it is more likely to reduce the duration of mechanical ventilation, potentially leading to a shorter ICU length of stay. In Japan, ICU management costs approximately $920 per day, with ICU management expenses accounting for a significantly larger proportion of total costs than medication expenses. Therefore, utilizing RF to reduce the duration of mechanical ventilation may offer a cost-effective advantage by potentially decreasing ICU length of stay.

Although this report includes only two cases, it demonstrates that RF can be effectively used for analgesia in intubated, mechanically ventilated pediatric patients requiring deep sedation in the ICU. RF administration before extubation may reduce the need for other sedatives and analgesics while maintaining appropriate sedation, enabling safe, early extubation.

## Conclusions

This report highlights the potential utility of RF as a safe and effective option for sedation and analgesia in pediatric patients undergoing mechanical ventilation in the ICU. The two presented cases demonstrate that RF's unique pharmacokinetics allow for rapid adjustment and predictable extubation timing while minimizing withdrawal symptoms associated with other sedatives and analgesics.

While RF is not currently approved for pediatric ICU use in Japan, these cases suggest that its off-label application can address challenges in sedation management and improve patient outcomes. Further studies and clinical trials are needed to confirm the safety and efficacy of RF in broader pediatric ICU populations, paving the way for potential regulatory approval and incorporation into standard practice.

## References

[1] Vet NJ, Ista E, de Wildt SN, van Dijk M, Tibboel D, de Hoog M (2013). Optimal sedation in pediatric intensive care patients: a systematic review. Intensive Care Med.

[2] Fong JJ, Sylvia L, Ruthazer R, Schumaker G, Kcomt M, Devlin JW (2008). Predictors of mortality in patients with suspected propofol infusion syndrome. Crit Care Med.

[3] Hughes MA, Glass PS, Jacobs JR (1992). Context-sensitive half-time in multicompartment pharmacokinetic models for intravenous anesthetic drugs. Anesthesiology.

[4] Ista E, van Dijk M, Gamel C, Tibboel D, de Hoog M (2008). Withdrawal symptoms in critically ill children after long-term administration of sedatives and/or analgesics: a first evaluation. Crit Care Med.

[5] Welzing L, Oberthuer A, Junghaenel S, Harnischmacher U, Stützer H, Roth B (2012). Remifentanil/midazolam versus fentanyl/midazolam for analgesia and sedation of mechanically ventilated neonates and young infants: a randomized controlled trial. Intensive Care Med.

[6] Santonocito C, Noto A, Crimi C, Sanfilippo F (2018). Remifentanil-induced postoperative hyperalgesia: current perspectives on mechanisms and therapeutic strategies. Local Reg Anesth.

[7] Akinci SB, Kanbak M, Guler A, Aypar U (2005). Remifentanil versus fentanyl for short-term analgesia-based sedation in mechanically ventilated postoperative children. Paediatr Anaesth.

[8] Naples J, Hall MW, Tobias JD (2016). Sedation with a remifentanil infusion to facilitate rapid awakening and tracheal extubation in an infant with a potentially compromised airway. J Pain Res.

[9] Kontou A, Agakidou E, Chatziioannidis I, Chotas W, Thomaidou E, Sarafidis K (2024). Antibiotics, analgesic sedatives, and antiseizure medications frequently used in critically ill neonates: a narrative review. Children (Basel).

[10] (2024). DRUG: remifentanil hydrochloride. https://www.kegg.jp/entry/D01177.

[11] Sakiko U, Taiga I, Katsumi S, Yushi A, Yukako O, Shigehito S (2008). Remifentanil infusion for dressing changes in a pediatric patient with burns: a case report [Article in Japanese]. J Clin Anesth (Japan).

[12] Goudra BG, Singh PM, Manjunath AK (2014). Effectiveness of high dose remifentanil in preventing coughing and laryngospasm in non-paralyzed patients for advanced bronchoscopic procedures. Ann Thorac Med.

[13] (2025). Remifentanil intravenous injection [Website in Japanese]. https://www.info.pmda.go.jp/go/pack/8219401D1030_1_05/.

[14] Ross AK, Davis PJ, Dear Gd GL (2001). Pharmacokinetics of remifentanil in anesthetized pediatric patients undergoing elective surgery or diagnostic procedures. Anesth Analg.

[15] Welzing L, Link F, Junghaenel S, Oberthuer A, Harnischmacher U, Stuetzer H, Roth B (2013). Remifentanil-induced tolerance, withdrawal or hyperalgesia in infants: a randomized controlled trial. RAPIP trial: remifentanil-based analgesia and sedation of paediatric intensive care patients. Neonatology.

[16] Büttner W, Finke W (2000). Analysis of behavioural and physiological parameters for the assessment of postoperative analgesic demand in newborns, infants and young children: a comprehensive report on seven consecutive studies. Paediatr Anaesth.

[17] Joly V, Richebe P, Guignard B, Fletcher D, Maurette P, Sessler DI, Chauvin M (2005). Remifentanil-induced postoperative hyperalgesia and its prevention with small-dose ketamine. Anesthesiology.

[18] Angst MS, Clark JD (2006). Opioid-induced hyperalgesia: a qualitative systematic review. Anesthesiology.

